# Supplementary material for: Asynchronous magnetic resonance elastography: Shear wave speed reconstruction using noise correlation of incoherent waves
Source: Magn Reson Med. 2022 Oct 27;89(3):990–1001. doi: 10.1002/mrm.29502 (PMC9792433; doi:10.1002/mrm.29502)
Supplement: Supplementary file 1 — DATA S1 MATLAB code used to generate the numerical simulations of Figure 1 [file MRM-89-990-s002.zip › k-Wave/helpfiles/example_sd_sensor_directivity_2D.html]

Sensor Element Directivity In 2D Example (k-Wave)


# Sensor Element Directivity In 2D Example

This example shows how to attribute a directional response to a single-element sensor, or to individual elements of a multi-element sensor array. Directionality can be included without a separate function through explicit averaging, as shown in the examples Modelling Sensor Directivity In 2D and Focussed Detector In 2D, but the functionality described here allows greater flexibility. Note, that directivity defined in this way is currently only supported in 2D. This example builds on the Homogeneous Propagation Medium and Using A Binary Sensor Mask examples.

- Open the file in the MATLAB Editor
- Run the file in MATLAB

## Contents

- Defining directional detectors
- Running the simulation (initial value problem)
- Running the simulation (time-varying source)

## Defining directional detectors

When using a binary sensor mask, the directionality of each sensor element (defined by the 1's within `sensor.mask`) can be set by assigning a directivity angle to the corresponding elements within an `Nx` by `Ny` matrix assigned to `sensor.directivity_angle`. This angle defines the direction of greatest sensitivity for each element. For waves incident from other angles, the sensitivity will be reduced. In this example, a line of spaced sensor points is assigned to a binary sensor mask with each given a different directionality. A directivity angle of 0 (or pi) results in the element being most sensitive to waves travelling in the x (up/down) direction. A directivity of pi/2 (or -pi/2) results in the element being most sensitive to waves travelling in the y (left/right) direction. Figure-of-eight (cos(theta)) directionality can be selected by setting `sensor.directivity_pattern = 'gradient'`. With this setting, waves incident at right angles to the sensor element will not be detected. Alternatively, the directionality can be chosen to be equivalent to spatial averaging by setting `sensor.directivity_pattern = 'pressure'`. In this case, it is also necessary to set the sensor element size using `sensor.directivity_size`. For a plane wave incident on a parallel linear detector, this value is equivalent to the size of sensor that each element averages across. If this field is not set, it defaults to `10 * kgrid.dx` (i.e., 10 times the width of the spacing between grid points).

```
% define a line of sensor points
sensor.mask = zeros(Nx, Ny);
sensor.mask(24, 2:2:63) = 1;

% define the angle of max directivity for each sensor point:
%    0             = max sensitivity in x direction (up/down)
%    pi/2 or -pi/2 = max sensitivity in y direction (left/right)
dir_angles = (-1:1/15:1).' * pi/2;

% assign to the directivity mask
sensor.directivity_angle = zeros(Nx, Ny);
sensor.directivity_angle(sensor.mask == 1) = dir_angles;

% define the directivity pattern
sensor.directivity_pattern = 'pressure';

% define the directivity size
sensor.directivity_size = 16 * kgrid.dx;
```

A visualisation of the sensor mask showing the directivity of each element is shown below, superimposed over an image of the source. Note that having many different directivity angles in a single simulation can increase the computation time. However, arrays in which all the elements have the same directionality will run almost as fast as the default omni-directional case.


## Running the simulation (initial value problem)

The initial pressure distribution is set up in the usual way and the computation is invoked by calling `kspaceFirstOrder2D` with the inputs defined above. To ensure the plane wave source is not distorted by the perfectly matched layer, the part of the PML perpendicular to the propagation direction is turned off by setting the optional input `'PMLAlpha'` to `[2, 0]` (see Controlling The Absorbing Boundary Layer).

```
% define the initial pressure distribution
source.p0 = zeros(Nx, Ny);
source.p0(39:41, :) = 2;
 
% turn off the PML in the y-direction
input_args = {'PMLAlpha', [2, 0]};

% run the simulation
sensor_data1 = kspaceFirstOrder2D(kgrid, medium, source, sensor, input_args{:});
```

The maxima of the time series recorded by each sensor element are plotted below. It is clear that the elements with their directivity aligned to the plane wave exhibit greater sensitivity.


## Running the simulation (time-varying source)

It is interesting to run this example with a single frequency source, rather than the broadband source produced by the initial pressure distribution. A single frequency time-varying source plane wave source can defined as:

```
% define a time varying sinusoidal source (instead of an initial pressure)
source_freq = 12e6;     % [Hz]
source_mag = 0.25;      % [Pa]
source.p = source_mag * sin(2 * pi * source_freq * kgrid.t_array);

% define source mask and force to be binary
source.p_mask = source.p0;
source.p_mask(source.p_mask ~= 0) = 1; 

% remove initial pressure field
source = rmfield(source, 'p0');
```

The computation is invoked by calling `kspaceFirstOrder2D` with the inputs defined above. As above, to ensure the source remains as a plane wave, is is necessary to turn off the PML along the sides perpendicular to the wavefront. The maxima of the time series recorded by each sensor element are plotted below on Cartesian and polar plots. Because this example uses a single frequency, the characteristic side lobes associated with spatial averaging detectors can be seen.
